# Video-assisted thoracoscopic surgery and thoracotomy during lobectomy for clinical stage I non-small-cell lung cancer have equivalent oncological outcomes: A single-center experience of 212 consecutive resections

**DOI:** 10.3892/ol.2014.2804

**Published:** 2014-12-17

**Authors:** CHUNHUA LIU, ZHONGDONG LI, CUIQING BAI, LI WANG, XUEFEI SHI, YONG SONG

**Affiliations:** 1 Department of Respiratory Medicine, Jinling Hospital, Nanjing University School of Medicine, Nanjing, Jiangsu 210002, P.R. China; 2 Department of Cardiothoracic Surgery, Jinling Hospital, Nanjing University School of Medicine, Nanjing, Jiangsu 210002, P.R. China; 3 Department of Respiratory Medicine, Nanfang Hospital, Southern Medical University, Guangzhou, Guangdong 510515, P.R. China

**Keywords:** video-assisted thoracoscopic surgery, thoracotomy, non-small cell lung cancer, mediastinal lymph node, disease-free survival, overall survival

## Abstract

The aim of the present study was to compare the oncological outcomes following lobectomy using either video-assisted thoracoscopic surgery (VATS) or thoracotomy in clinical stage I non-small cell lung cancer (NSCLC) patients. Short- and long-term data from 212 consecutive patients who underwent lobectomy for clinical stage I NSCLC via VATS or thoracotomy between February 2003 and July 2013 were retrospectively reviewed. The primary endpoints were mediastinal lymph node staging, disease-free survival time and overall survival time. A total of 212 lobectomies for clinical stage I NSCLC were performed, 123 by VATS and 89 by thoracotomy. Patients’ demographic data, pathological stage and residual tumor were similar in the two groups. Reduced blood loss, less post-operative analgesia required and earlier hospital discharge were recorded for the VATS group, as compared with the thoracotomy group. The overall morbidity was similar in the two groups. However, the rate of major complications was higher following thoracotomy than following VATS. No 30-day mortality occurred subsequent to either thoracotomy or VATS lobectomy. The overall survival and disease-free survival times were comparable between the two groups. In the univariate analysis, the treatment approach was not associated with the overall five-year survival or the disease-free survival times. Multivariate Cox regression analysis of survival times revealed that significant predictors of shorter survival times were advanced pathological T3 stage, pathological N1 or N2 disease and poor cancer differentiation. In conclusion, it is reasonable to conclude from the present study that VATS lobectomy performed by specialist thoracic surgeons is safe and may achieve similar long-term survival times to the open surgery approach. However, further prospective randomized multi-center trials are warranted prior to incorporating VATS into clinical routine.

## Introduction

Lung cancer remains the leading cause of cancer morbidity and mortality worldwide (1,2). Lobectomy by thoracotomy is recognized as a primary procedure in the treatment of early-stage non-small cell lung cancer (NSCLC) (3–6). Although lobectomy via thoracotomy provides optimal locoregional control and long-term survival, this procedure is associated with high mortality and morbidity rates: 2–10 and 30–50%, respectively (2–6). Therefore, the identification of alternative techniques that diminish surgical trauma without compromising oncological outcome is required. Jacobaeus first performed thoracoscopy for inspecting the pleural space in 1910 (7). Until the 1980s, thoracoscopy was only employed for diagnosis of pleural diseases. However, after McKenna reported his initial experiences of 44 patients who had undergone video-assisted thoracoscopic surgery (VATS) in 1994 (8), major advances in VATS, such as VATS lobectomy and VATS segmentectomy, were achieved in the late 1990s (9). With the worldwide employment of VATS, reduced postoperative trauma and postoperative morbidity has been reported (10–12). Certain surgeons employ VATS lobectomy to reduce surgical trauma. However, the oncological outcomes following VATS lobectomy, as measured using mediastinal lymph node dissection and long-term survival times, have not been fully elucidated (9,13–19). In addition, few multi-center randomized controlled trials that compare the two approaches and the long-term oncological outcomes have been conducted (16–19).

VATS lobectomy for clinical stage I NSCLC was introduced to Jingling Hospital (Nanjing, China) in January 2008. The thoracic surgeons in the Department of Thoracic Surgery of the hospital have the basic ability to perform VATS. The present study aimed to assess the oncological outcomes following VATS lobectomy by reviewing five years of experience performing VATS lobectomy at the hospital.

## Patients and methods

### Patient evaluation

The present retrospective study complied with the Declaration of Helsinki rules and was approved by the Ethics Committee of Jinling Hospital (Nanjing, China). The requirement for informed consent from all patients was waived due to the retrospective nature of the study.

Data from 212 consecutive patients with clinical stage I NSCLC who underwent lobectomy at the Department of Thoracic Surgery, Nanjing General Hospital of Nanjing Military Command (Nanjing, China) between February 2003 and July 2013 were retrospectively reviewed. All patients underwent bronchoscopy, endobronchial ultrasound, and computed tomographic scans of the brain, chest and upper abdomen prior to surgery. Mediastinoscopy was not required except when positive mediastinal or hilar lymph nodes were detected using the chest computed tomographic scan. Positron emission tomography-computerized tomography (CT) and bone scanning were performed on all patients. Classification of the tumor clinical stage was determined by the 7th edition of the TNM classification of lung cancer (20), which was proposed by the Union for International Cancer Control (UICC) and the International Association for the Study of Lung Cancer (IASLC). The mediastinal lymph node staging was determined by the most recent lymph node map proposed by the IASLC (21). For those patients who underwent surgery prior to 2009, staging was recalculated to match the 7th edition of TNM classification of lung cancer proposed by UICC and IASLC (21).

### Surgical technique

All surgical procedures, including VATS and thoracotomy, were performed by three senior surgeons with proven expertise in lung cancer, who had conducted >60 VATS lobectomies and >300 open lobectomies prior to the present study. The selection of VATS or open lobectomy was decided by the patients and their families. The resection was considered to be administered with curative intent (R0) in all cases. Only trocars and endsocopic instruments were used in the VATS lobectomies and no rib spreading was performed. All patients underwent one-lung ventilation and were placed in the lateral decubitus position. Mediastinal lymph node dissection was routinely performed. We did not perform an intra-operative lymph node frozen section analysis, due to the time-consuming nature of the procedure.

On the left side, the 5, 6, 7, 8, 9, 10, 11 and 12 lymph node stations, and on the right side, the 2R, 4R, 7, 8, 9, 10, 11 and 12 lymph node stations were systematically dissected *en bloc*. The lymph nodes were dissected with the integrity of the surrounding structure, with clear recognition of the anatomical landmarks and with no nodal structures. Lymph node stations 10, 11 and 12, and the affected lobes were systematically dissected. On the right side, when the mediastinal pleura were opened, stations 2R and 4R was dissected up to the lowest visible section of the subclavian artery. After the lung was retracted anteriorly, station 7 dissection was performed. Regardless of middle lobectomy and lower lobectomy, stations 8 and 9 were systematically harvested. On the left side, stations 5 and 6 were systematically harvested. Subsequently, dissection of stations 7, 8 and 9 was performed (16).

### Surgical outcome and post-operative complications

The operative time, degree of blood loss, pathological stage, overall number of lymph nodes dissected, residual tumor status, post-operative morbidity occurring within 30 postoperative days and length of hospital stay were assessed. The morbidity occurring within 30 postoperative days, which included major and minor complications, was graded according to the Clavien-Dindo classification (22). Major complications were defined as grades 3b, 4a, 4b and 5. Minor complications were classified as grades 1, 2 and 3a. The 30-day mortality was defined as all-cause fatality within 30 postoperative days.

### Follow-up

During the first year following treatment, the patients were assessed every three months at the outpatient department. In the second year, follow-up was conducted every six months and, subsequently, follow-up was performed at the end of each year. During follow-up, diagnostic investigations were performed. All patients received CT chest scans prior to discharge and prior to each follow-up visit. Any post-operative complications and medical conditions that required hospitalization were reviewed. The final follow-up assessment occurred in November 2013.

### Statistical analysis

For statistical analysis, SPSS 13.0 for Windows (SPSS, Inc., Chicago, IL, USA) was used. Data are presented as the mean ± standard deviations for variables following a normal distribution, and were analyzed by Student’s t-test. For variables following a non-normal distribution, the results are expressed as the median and range, and were compared by nonparametric test. Differences of semi-quantitative results were analyzed by the Mann-Whitney U test. Differences of qualitative results were analyzed by the χ^2^ or Fisher exact test, where appropriate. The survival rates were analyzed using the Kaplan-Meier method; the differences between the two groups were analyzed with the log-rank test. The overall survival time was classified as the time period between the date of surgery and the date of the final follow-up or fatality from any cause. The disease-free survival time was calculated as the time period between the date of surgery and the date of cancer recurrence or fatality from any cause. Univariate analysis was performed to identify prognostic variables associated with overall survival time. Univariate variables with P<0.05 were selected for inclusion in the multivariate Cox proportional-hazards regression model. The adjusted odds ratios along with the corresponding 95% confidence intervals were calculated. P<0.05 was considered to indicate a statistically significant difference.

## Results

### Demographic data

The demographic data are summarized in Table I. During the present study, 123 lobectomies by VATS and 89 lobectomies by thoracotomy were performed. No significant differences were identified in age, gender, comorbidity, forced expiratory volume in the first second (observed to predicted), tumor size, clinical stage, number of mediastinoscopies and American Society of Anesthesiologists Physical Status classification system score (23) between the two groups (P>0.05).

### Surgical outcome and pathological data

The surgical and pathological outcomes are summarized in Table II. No conversion to open lobectomy occurred in the cases where VATS was performed. No intraoperative or in-hospital mortality occurred in either group. Significantly longer operative times were recorded in the VATS group as compared with the open surgery group (P<0.05). No significant differences in pathological stage or residual tumor status between the two groups was identified (P>0.05). Patients in the VATS group exhibited significantly faster recovery, with reduced blood loss (P<0.05), less post-operative analgesia required (P<0.05) and earlier hospital discharge (P<0.05) than the patients who had undergone thoracotomy.

### Lymph nodes and stations harvested

The nodes and stations harvested, including N1 and N2, are summarized in Table III. No significant differences between the two groups were detected when comparing either the number of lymph node stations or the overall number of lymph nodes dissected (P>0.05) The numbers of harvested lymph nodes and lymph node stations were also similar in the two groups (P>0.05). The number of harvested lymph nodes in each resection was >10 and a minimum of six lymph node stations from each patient were harvested. The lymphadenectomy results were comparable with those of other studies (Table IV).

### Post-operative complications

The post-operative complications in the VATS and thoracotomy groups are reviewed in Table V. The overall morbidity within 30 postoperative days was similar in the two groups (P>0.05). However, when the severity of complications was compared, a significantly greater number of complications were classified as major in patients who had undergone thoracotomy, as compared with the VATS patients (P<0.05).

### Overall survival

**Figure 1:**
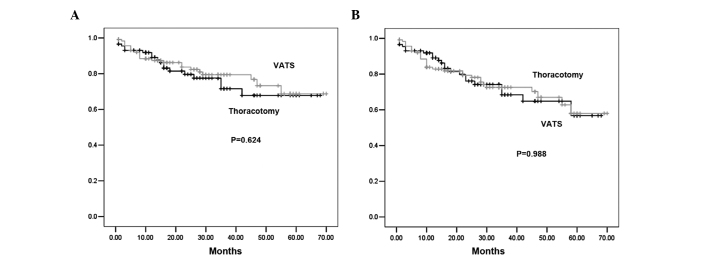
(A) Overall survival and (B) disease-free survival in association with lobectomy approach (VATS vs. thoracotomy) in 212 consecutive patients. VATS, video-assisted thoracoscopic surgery.

The median follow-up duration was 36 months, with similar average follow-up times in the two groups. No differences in overall survival times between the VATS and open surgery groups were identified (P=0.624; Fig. 1). The three- and five-year overall survival rates were 79.2 and 71.6%, respectively, in the VATS group as compared with 72.6 and 68.0%, respectively, in the thoracotomy group. Multivariate Cox regression analysis of the overall survival times of all patients in the whole cohort was also performed. Significant predictors of shorter overall survival times were T3 pathological stage (P=0.001), pathological N1 or N2 disease (P=0.001), and poor tumor differentiation (P=0.005) (Table VI). The VATS surgical approach was not found to be a significant predictor for overall survival time by univariate analysis.

### Disease-free survival

When the disease-free survival rates were examined, the three- and five-year disease-free survival rates were 75.3 and 59.0%, respectively, in the VATS group as compared with 70.1 and 58.2%, respectively, in the thoracotomy group, with no statistically significant differences identified between the two groups (P=0.988; Fig. 1). To determine whether the patients who underwent VATS exhibited a higher incidence of recurrent cancer as compared with the open surgery patients, recurrence patterns and time to recurrence were examined (Table VII). The location of the recurrence and the time to recurrence were not significantly different between the two groups. No port-site recurrence was noted in the VATS cases. Multivariate Cox regression analysis of disease-free survival times revealed that significant predictors of shorter disease-free survival times were advanced pathological T3 stage (P=0.023), pathological N1 or N2 disease (P=0.003) and poor tumor differentiation (P=0.020) (Table VIII). The surgical approach was not found to be a significant predictor for reduced disease-free survival times. The oncological outcomes were comparable with those of other large sample size studies (Table IX).

## Discussion

Although VATS lobectomy for stage I NSCLC has been widely used due to proven benefits, the merits of the technique with regard to oncological outcomes remains controversial (24–27). According to the Society of Thoracic Surgeons database (28), only 20% of lobectomies are performed via VATS, with 80% conducted using conventional thoracotomy. The success of VATS can only be definitively measured using the long-term survival times, as compared with those following thoracotomy. In the present study, VATS lobectomy and thoracotomy lobectomy were compared using a consecutive series of patients who underwent surgery performed by surgeons extensively experienced in VATS and open lobectomies. The study demonstrated that VATS lobectomy achieves similar oncological results to conventional thoracotomy.

Although previous studies have been conducted regarding the effect of mediastinal lymph node removal and systematic mediastinal lymph node dissection on long-term survival times (29,30), there remains controversy with regard to the impact of lymphadenectomy on oncological outcome (31). The largest prospective randomized control trial comparing mediastinal lymph node sampling with dissection, termed the Z0030 trial, was reported by the American College of Surgery Oncology Group (29). This trial revealed that mediastinal lymph node dissection achieved similar long-term survival times as compared with lymph node sampling in early-stage NSCLC patients without evidence of mediastinal or hilar lymph node metastasis confirmed by sampling. Therefore, the result of the Z0030 trial is only suitable for highly selected NSCLC patients and is not applicable for all operable NSCLC patients. Since producing intra-operative frozen sections is time-consuming and the results of the Z0030 trial are only applicable for particular patients, intra-operative lymph node staging is not performed in China. However, in a prospective randomized control trial involving 532 patients with clinical stage I–IIIA NSCLC, 268 patients underwent mediastinal lymph node dissection and 264 patients underwent mediastinal lymph node sampling performed by Chinese surgeons (30). The five-year survival rate in the patients who had undergone mediastinal lymph node dissection was significantly higher than that in those who had mediastinal lymph node sampling performed (P<0.05), regardless of the clinical stage. Thus, systematic mediastinal lymph node dissection was routinely performed for all operable NSCLC patients in the present study cohort.

The quality of mediastinal lymph node dissection is the core component when VATS lobectomy is performed. The majority of studies previously reported have revealed no differences in the quality of mediastinal lymph node dissection during VATS as compared with thoracotomy (16–19). However, certain studies have reported the quality of lymph node dissection to be inferior to that of thoracotomy, particularly in the early stages of VATS lobectomy (31–33). The CALGB 39802 study (32), a prospective multi-center study, observed that over half of the resections in patients undergoing VATS had fewer than two stations sampled and ~15% resections in patients undergoing VATS had no lymph nodes harvested. The quality of lymphadenectomy in the CALGB 39802 study was far from the recommendation proposed by IASLC that a minimum of six lymph node stations be removed or sampled in lobectomy (31,34). IASLC also recommends that three of these lymph node stations be mediastinal lymph nodes (31,34). The results from the present study revealed that the number of dissected lymph node stations and lymph nodes were similar in VATS and thoracotomy, and that the quality of lymphadenectomy was applied with recommendation proposed by IASLC.

An increasing number of studies have reported similar perioperative outcomes following VATS and open lobectomy (32,35). The results from the present study did not differ from the conclusions drawn from other studies; VATS lobectomy was safe and less trauma occurs, as compared with thoracotomy (16–19). In the present study, the NSCLC patients who underwent VATS lobectomy benefited from a quicker recovery, with less bleeding, reduced post-operative pain, shorter duration of chest drainage and earlier hospital discharge as compared with the patients who underwent thoracotomy lobectomy, and these findings were comparable with those of previous studies (36,37).

The long-term outcomes, measured by overall survival and disease-free survival times, were comparable with those of other studies concerning VATS versus open lobectomy (24–27,37). As thoracic surgeons became more skillful with VATS and recognized the benefits, such as shorter recovery times and reduced trauma, establishing prospective randomized multi-center trials that compared VATS and thoracotomy was difficult, due to difficulty in enrolling patients on a long-term basis, as VATS have a short-term outcome when compared with open resection. Studies regarding the long-term outcomes of VATS versus open lobectomy are mainly retrospective and produce prospective non-randomized comparisons (24–27,36). These studies have revealed marginally improved overall survival and disease-free survival times following VATS (24–27,37). In the present study, the patients who had undergone VATS exhibited marginally improved survival times and later recurrence than the patients who had thoracotomy performed. This finding may be difficult to explain. Chen *et al* (38) have hypothesized that the reduced trauma during VATS lobectomy may result in quicker recovery time, earlier administration of adjuvant chemotherapy and improved compliance with adjuvant chemotherapy. The other factor may be that the reduced immunological suppression during VATS as compared with thoracotomy increases the patient’s ability to scavenge residual cancer cells shed into the blood or lymphatics at lobectomy (36,38–40). However, the underlying mechanisms for this process require further investigation.

Certain limitations of the present study must be acknowledged. The study was based at a single center, not at multiple centers, and the results were produced from retrospective analysis, not prospective randomized analysis. Therefore, bias in the selection of patients and the surgical approach, by the surgeons, cannot be excluded. This limitation needs accounting for when interpreting the results. Other factors that may affect long-term outcomes and prognosis, such as adjuvant therapy and treatment for recurrence and distant metastasis, are not completely described by this analysis.

In summary, it is reasonable to conclude from the present study that VATS lobectomy performed by specialist thoracic surgeons is safe and achieves similar long-term survival times to the open surgery approach. However, further prospective randomized multi-center trials are warranted prior to the incorporation of VATS into clinical routine practice.

## Figures and Tables

**Table I:** Demographic data.

Parameter	VATS (n=123)	Thoracotomy (n=89)	P-value
Age, years^a^	65.0 (50–70)	64.0 (46–75)	0.859
Gender, male:female	74:49	51:38	0.676
Comorbidity, n			0.373
COPD	2	1	
Hypertension	12	8	
Diabetes Mellitus	6	2	
Smoking	46	32	
Atrial fibrillation	1	2	
Earlier myocardial infarction	1	2	
FEV1 (observed to predicted), %^b^	86.0 (76–95)	86.0 (80–98)	0.920
Tumor size, cm^b^	1.90 (0.8–3.9)	1.90 (0.5–3.9)	0.675
Clinical stage, n			0.750
IA	65	49	
IB	58	40	
Mediastinoscopy, n	2	3	0.713
ASA score, n			0.546
I	66	42	
II	59	45	
III	1	2	

a Median (range) and

b mean (± SD).

VATS, video-assisted thoracoscopic surgery; COPD, chronic obstructive pulmonary disease; FEV1, forced expiratory volume in the first second; ASA, American Society of Anesthesiologists.

**Table II:** Surgical and pathological data.

Clinical parameter	VATS (n=123)	Thoracotomy (n=89)	P-value
Type of resection, n			0.305
Left upper lobectomy	36	16	
Left lower lobectomy	24	20	
Right upper lobectomy	21	13	
Right middle lobectomy	10	8	
Right lower lobectomy	32	32	
Operative time, min^a^	200.0 (120–380)	160.0 (100–330)	0.000
Blood loss, ml^a^	160.0 (100–320)	210.0 (110–500)	0.000
Histological type, n			0.166
Adenocarcinoma	101	81	
Squamous cell carcinoma	20	7	
Other	2	1	
Pathological stage, n			1.000
IA	42	26	
IB	64	51	
IIA	10	7	
IIB	5	3	
IIIA	2	2	
Residual tumor, R0/R1/R2, n	122/1/0	87/2/0	0.384
Post-operative analgesia, days^b^	2.0 (1.0–5.0)	5.0 (1.0–6.0)	0.000
Duration of chest drainage, days^b^	6.0 (3–9)	7.0 (5–13)	0.000
Hospital stay, days^b^	8.0 (6–21)	16.0 (11–21)	0.000

a Median (range) and

b mean (± SD).

VATS, video-assisted thoracoscopic surgery; COPD, chronic obstructive pulmonary disease; FEV1, forced expiratory volume in the first second; ASA, American Society of Anesthesiologists.

**Table III:** Nodes and stations harvested.

Parameter^a^	VATS (n=123)	Thoracotomy (n=89)	P-value
No. of harvested lymph node stations	8.0 (6–8)	8.0 (6–8)	0.449
No. of mediastinal lymph node stations dissected	5.0 (3–5)	5.0 (3–5)	0.344
No. of harvested lymph nodes	28.0 (22–36)	28.0 (22–40)	0.164
No. of mediastinal lymph nodes dissected	17.0 (12–23)	17.0 (12–28)	0.110

a Median (range).

VATS, video-assisted thoracoscopic surgery.

**Table IV:** Literature review of mediastinal lymph node dissection using VATS vs. thoracotomy.

Study	No. patients	Lymph nodes	N1 lymph nodes	N2 lymph nodes	Lymph node stations
Palade *et al* (18) (2013, Germany)	VATS: 32	25.1	10.5	NR	NR
	Open: 32	25.2	8.9		
Yang *et al* (16) (2013, China)	VATS: 31	28.2	9.5	18.6	6.8
	Open: 31	29.8	8.4	21.4	6.7
Ramos *et al* (15) (2012, France)	VATS: 96	22.6	NR	17.7	5.1
	Open: 200	25.4		18.2	4.5
Watanabe *et al* (17) (2005, Japan)	VATS: 191	33.8	NR	23.4	NR
	Open: 159	30.9		21.0	

Lymph node data are presented as the means. VATS, video-assisted thoracoscopic surgery; NR, not reported.

**Table V:** Post-operative complications.

Adverse event	VATS (n=123)	Thoracotomy (n=89)	P-value
Post-operative complications, n	31	21	0.763
Severity of complications, n			0.002
Major (3b, 4a, 4b or 5)	4	11	
Minor (1, 2 or 3a)	27	10	
Major, n			1.000
Pulmonary embolism	1	3	
Acute coronary syndrome	1	2	
Respiratory insufficiency	1	4	
DIC	1	2	
Minor, n			1.000
Pneumonia	6	2	
Wound infection	3	1	
Urinary tract infection	4	1	
Atrial fibrillation	5	1	
Chylothorax	3	1	
Recurrent nerve palsy	3	1	
Prolonged air leak (>5 days)	3	3	
Mortality within 30 days after surgery, n	0	0	

Lymph node data are presented as the means. VATS, video-assisted thoracoscopic surgery; NR, not reported.

**Table VI:** Multivariate Cox regression analysis of overall survival times.

Regression variable	Adjusted hazard ratio	95% CI	P-value
Pathological T stage			
T1	1.00		
T2	1.23	0.51–2.36	0.896^a^
T3	2.36	1.52–5.69	0.001^a^
Pathological N stage			
N0	1.00		
N1/N2	1.23	0.65–3.65	0.001^b^
Differentiation grade			
Good	1.00		
Moderate	1.36	0.36–2.36	0.259^c^
Poor	3.25	1.23–6.89	0.005^c^

a Compared with T1;

b compared with N0;

c compared with differential grade ‘good’.

**Table VII:** Comparison of recurrence pattern and site following lobectomy.

Recurrence parameter	VATS	Thoracotomy	P-value
Overall recurrence, n (%)	15 (12.1)	12 (13.5)	1.000
Locoregional, n (%)	8 (6.5)	7 (7.9)	1.000
Mediastinal lymph node	2	1	
Pleura	2	3	
Ipsilateral lung	4	3	
Distant, n (%)	7 (5.6)	5 (5.6)	1.000
Brain	3	2	
Liver	2	2	
Bone	1	1	
Median time to recurrence, months	18	16	0.360

VATS, video-assisted thoracoscopic surgery.

**Table VIII:** Multivariate Cox regression analysis of disease-free survival times.

Regression variable	Adjusted hazard ratio	95% CI	P-value
Pathological T stage			
T1	1.00		
T2	1.36	0.63–2.69	0.450
T3	3.20	1.56–4.62	0.023
Pathological N stage			
N0	1.00		
N1/N2	2.31	0.96–4.21	0.003
Differentiation grade			
Good	1.00		
Moderate	1.62	0.25–3.22	0.230
Poor	3.58	1.69–6.32	0.020

**Table IX:** Literature review of long-term survival rates following VATS or thoracotomy.

				Overall survival rate (%)		Disease-free survival rate (%)	
					
Study	Clinical stage	Approach	No.	Three-year	Five-year	Three-year	Five-year
Lee *et al* (23) (2013, USA)	I	VATS	188	87.4	76.5	77.7	61.1
		Open	187	81.6	77.5	76.9	72.1
Thomas *et al* (24) (2002, France)	I	VATS	110	NR	62.9	NR	NR
		Open	404	NR	62.8	NR	NR
Shiraishi *et al* (25) (2006, Japan)	I	VATS	81	NR	89.1	NR	79.0
		Open	79	NR	77.7	NR	80.2
Flores *et al* (26) (2009, USA)	I	VATS	398	NR	79.0	NR	NR
		Open	343	NR	75.0	NR	NR

VATS, video-assisted thoracoscopic surgery; NR, not reported.
